# MED21/393: ICD as a Search Tool for Medical Internet Resources

**DOI:** 10.2196/jmir.1.suppl1.e63

**Published:** 1999-09-19

**Authors:** K Adelhard, J Eissner

**Affiliations:** ^1^IBE - Medical Informatics, Biometry Epidemiology, MünchenGermany

**Keywords:** Medical Informatics Applications, Medical Record Linkage

## Abstract

**Introduction:**

The Internet offers information on specific diseases on WWW-server distributed over the entire world. Selecting the appropriate information resource is a non-trivial task. Several approaches (like MeSH, MedPix) exist to describe the contents of Web pages. However, the majority of the Web sites do not make use of these schemas. Automatic (or semi-automatic) linking of medical records to information resources on the internet is not sufficiently supported. ICD (International Classification of Diseases) is the standard for coding the diagnosis in medical records. The purpose of this study was to evaluate the feasibility of ICD - based search tools.

**Methods:**

A general ICD Meta-Search Engine (ICD-Search) was established to perform ICD-based Internet searches. From the records of the Großhadern University Hospital, the 20 most frequent diagnosis were selected. The diseases are coded with ICD-9 by the medical personnel of the hospital. The English version of the ICD-9 was chosen for performing the internet study. General search engines (Yahoo, Lycos, AltaVista) were used to retrieve medical information corresponding to the selected ICD codes. The first 50 hits were included in the study. The Web pages were scored according to accessibility (information could be retrieved) of the Web pages and the contents (reflects ICD code). The quality of information (current state of knowledge, complete information, comprehensive presentation) was not take into account. Also the results of the different search engines were compared. This study was repeated with specialized medical search engines (MedHunt) . Scoring of the results was performed accordingly.

**Results:**

Both Lycos and AltaVista searched resulted in a large number of hits. The range for AltaVista was between 114.440 (Coronary atherosclerosis) and 405.770 (Alcohol dependence syndrome). The Lycos search resulted in no categories and four sites (Coronary atherosclerosis) and one site for Alcohol dependence syndrome. MedHunt found 131 corresponding sites for Coronary atherosclerosis and 112 for Alcohol dependence syndrome. The median scores for Lycos and Altavista were in the same order with some extreme low scores for AltaVista on selected diseases. Lycos, in general, scored better than Lycos and AltaVista but show a low number of hits for several diseases. MedHunt produced both good scores and a sufficient number of hits. The results from Lycos and AltaVista were compared. It showed, that only 15% of the web pages were found by both search engines.

**Conclusion:**

All search engines found Internet sites that corresponded to the selected ICD-codes. The standard vocabulary provided by ICD proved to be a good basis for linking medical diagnosis with Internet Web sites. Specialized medical search engines perform better than general ones. Search engines, that also give information on the quality of information, offer additional value.

